# A chromosome 2 locus controls transient Agrobacterium-mediated GFP expression in rose petals

**DOI:** 10.1007/s00299-026-03961-z

**Published:** 2026-09-04

**Authors:** Ninh Hai Ho, Marcus Linde, Thomas Debener

**Affiliations:** 1 College of Biotechnology Forestry, Vietnam National University of Forestry, Hanoi, Vietnam; 2 Institute for Plant Genetics, Leibniz University Hannover, Hannover, Germany

**Keywords:** Transient expression, Genome-wide association study, Tetraploid rose, Host susceptibility, Green fluorescent protein, Ornamental biotechnology

## Abstract

**Key message:**

A major chromosome 2 region is associated with the genotype-dependent transient GFP expression after Agrobacterium infiltration of rose petals and provides markers for selecting permissive genotypes.

**Abstract:**

Transient expression assays are valuable for testing gene function in rose, where stable transformation remains laborious and genotype-dependent. We evaluated *Agrobacterium*-mediated transient green fluorescent protein (GFP) expression in the petals of 96 rose genotypes and used genome-wide association analysis to identify associated host genomic regions. GFP expression was scored semiquantitatively on a scale from 0 to 4 at 3 and 5 days post-infiltration (dpi). The genotypes differed strongly in expression at both time points, and the 3- and 5-dpi scores were strongly correlated (*r* = 0.90, *p* < 0.001). At 5 dpi, the mean scores ranged from 0 to 3.98, with the cultivars Sebastian Kneipp, Friesia and Comtessa AL among the most permissive genotypes. A genome-wide association study (GWAS) of 37,161 high-quality single-nucleotide polymorphisms (SNPs) revealed a major association on chromosome 2 at 69–73 Mbp and a second peak on unanchored chromosome 0 contigs that may correspond to the same region. The top SNPs reached -log10(P) = 7.85. This identified interval represents a high-priority candidate region containing multiple potential host factors. Candidate annotations within the region include genes related to auxin transport, ubiquitin-mediated protein turnover, ribonucleic acid (RNA) metabolism, membrane-associated defence responses and nuclear transport. The results identify useful rose genotypes for petal transient assays and provide a genetic entry point for dissecting host control of *Agrobacterium*-mediated transient expression.

**Supplementary Information:**

The online version contains supplementary material available at https://doi.org/10.1007/s00299-026-03961-z.

## Introduction

Roses are among the most economically important ornamental crops and are being increasingly supported by genomic resources for trait analysis, marker-assisted selection and functional genomics (Bendahmane et al. 2013; Hibrand Saint-Oyant et al. 2018; Raymond et al. 2018; Leus et al. 2018). For functional studies and precision breeding, however, roses remain technically challenging, because efficient transformation and regeneration are not available for all genotypes. This limitation restricts the routine validation of genes underlying traits, such as flower colour, scent, senescence, stress responses and plant architecture.

*Agrobacterium*-mediated gene transfer is a central tool in plant biotechnology, but its efficiency depends on a sequence of bacterial and host processes, including bacterial attachment, transfer-DNA (T-DNA) transfer, nuclear import, expression and, in stable transformation, integration (Tzfira and Citovsky 2006; Lacroix and Citovsky 2013; Hwang et al. 2017). The host genotype can substantially affect these processes. In *Brassica oleracea*, quantitative trait loci were identified for *Agrobacterium*-mediated transient and stable transformation (Cogan et al. 2004), indicating that transformation responsiveness can be treated as a genetically controlled trait. In rose, a recent GWAS of *Rhizobium rhizogenes*-mediated hairy root formation similarly emphasised the value of genetic mapping for transformation-related traits (Rüter et al. 2024).

Transient expression assays can partly bypass the time-consuming production of stable transgenic plants for some traits. Agroinfiltration in *Nicotiana benthamiana* and other species is widely used for rapid testing of promoters, reporters and candidate genes (Wroblewski et al. 2005; Lee and Yang 2006). In rose, *Agrobacterium*-based transient assays have been developed for petals and leaves (Yasmin and Debener 2010; Yasmin et al. 2013), and transient overexpression or silencing approaches have supported analyses of rose petal traits, such as abscission, dehydration tolerance and pigmentation (Gao et al. 2016; Zhang et al. 2017; He et al. 2023). Nevertheless, the genetic basis of genotype-dependent transient expression in rose petals has not been analysed at the genome scale.

Genome-wide association studies (GWASs) are particularly suitable for genetic analyses of traits measured in diverse germplasms, because they connect phenotypic variation to marker variation while accounting for relatedness and population structure (Yu et al. 2006; Alseekh et al. 2021). Rose GWAS has been applied to floral pigment traits, adventitious regeneration, callus formation, black spot resistance, adventitious rooting, marker-assisted selection of floral traits and hairy root formation (Schulz et al. 2016, 2023; Nguyen et al. 2017, 2020; Lopez Arias et al. 2020; Wamhoff et al. 2023; Rüter et al. 2024). The availability of the WagRhSNP 68K Axiom array and the *Rosa chinensis* reference genome enables marker-trait association analyses in tetraploid rose panels (Koning-Boucoiran et al. 2015; Hibrand Saint-Oyant et al. 2018).

Here, we used a 96-genotype rose association panel to quantify transient GFP expression in petals after *Agrobacterium* infiltration and to determine whether the observed variation has a genetic basis. The objectives of this study were to identify genotypes suitable for transient petal assays, determine whether major genomic regions control GFP expression, and identify candidate genes for future functional validation.

## Materials and methods

### Plant material and petal sampling

**Fig. 1:**
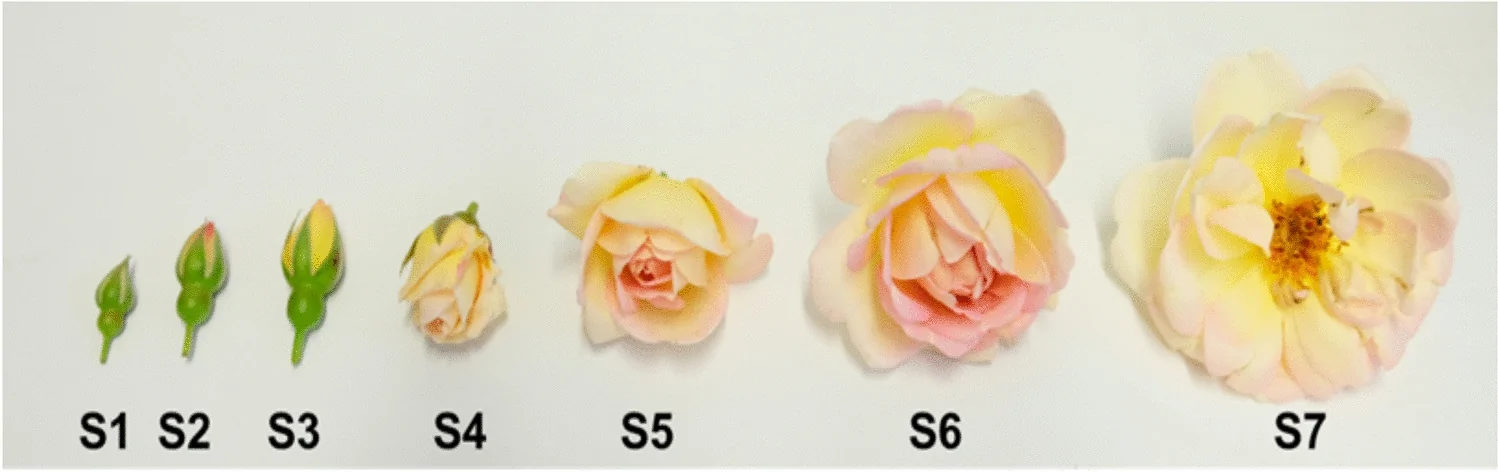
Rose flowers bloom through seven stages, from closed buds to fully open, senescent flowers. The seven stages of rose flower development are as follows: S1: unopened bud; S2: semi-open; S3: fully opened; S4: initial bloom; S5: full bloom; S6: bloomed; and S7: senescent

The association panel consisted of 96 mostly tetraploid cut and garden rose genotypes: 87 tetraploid, 8 triploid, and 1 diploid genotype. The plants were cultivated as potted plants under semi-controlled greenhouse conditions in three randomised blocks at the Federal Plant Variety Office (Bundessortenamt), Hannover, Germany, as described for this rose association panel in a previous GWAS (Schulz et al. 2016). For each genotype, 20 petals were harvested for each of the three biological replicates from flowers at developmental stages corresponding to initial and full bloom (Fig. 1, stages S4–S5), because these stages were previously shown to be suitable for rose petal agroinfiltration (Yasmin and Debener 2010). Petals from the outer whorl were removed, and only petals from the middle of the flower were used for infiltration.

### Bacterial strain, vector and infiltration

*Agrobacterium tumefaciens* strain GV3101 carrying the binary vector C757pGFPU10-35 s-ocs-LH (DNA cloning service, Hamburg, Germany) was used. The T-DNA contains an intron-containing GFP reporter driven by the *Arabidopsis* ubiquitin 10 promoter and bacterial selection markers for streptomycin and spectinomycin resistance (Online Resource 1, Fig. S1). GV3101 was grown on Yeast Extract Peptone (YEP) agar or in YEP liquid medium supplemented with rifampicin, streptomycin and spectinomycin (Online Resource 1; Table S1). A single colony was inoculated into 10 mL of liquid YEP and incubated overnight at 28 °C with shaking. The culture was refreshed 1:10 in pre-warmed YEP and grown to approximately OD600 = 1.5. The cells were collected by centrifugation, resuspended in sterile 5% sucrose solution, and adjusted to an OD600 of 1.0. The petals were wounded with a scalpel at the petal base and infiltrated from the abaxial side using a 1-mL needleless syringe. Infiltrated petals were placed in plastic boxes on moistened tissue paper and incubated at room temperature.

### Scoring of transient GFP expression

**Fig. 2:**
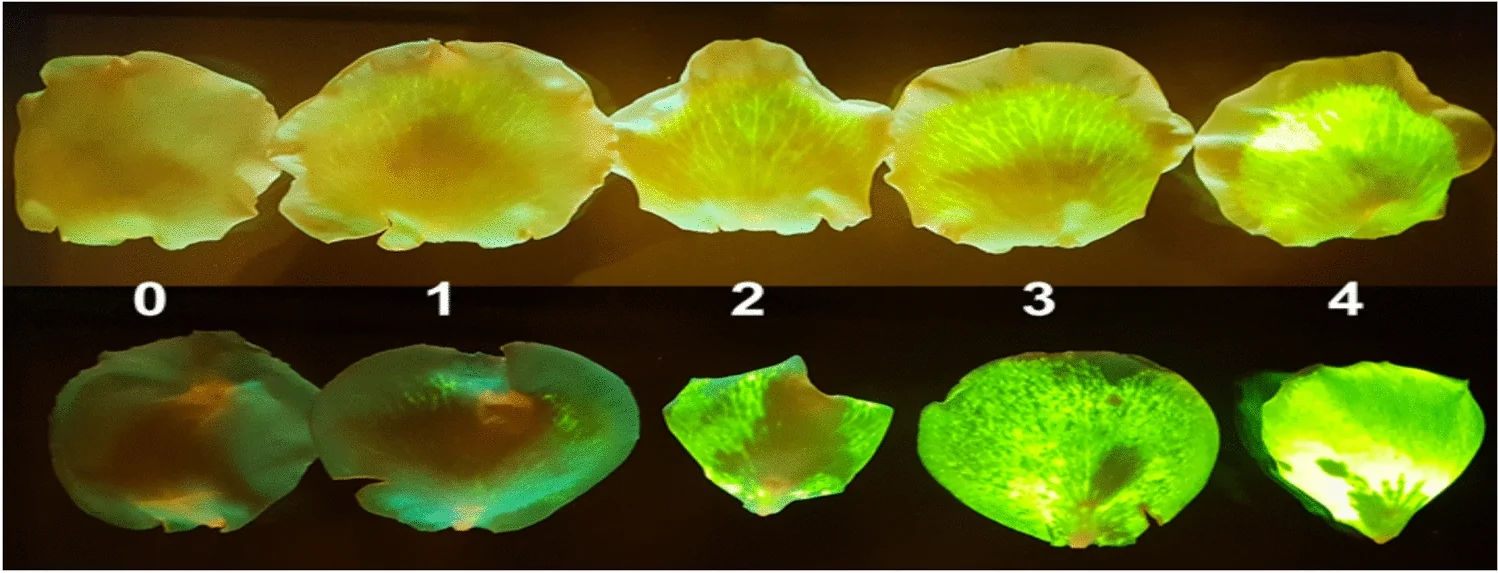
Semi-quantitative scoring of transient GFP expression in rose petals after Agrobacterium infiltration. Representative petals are shown for GFP expression classes 0–4 at 5 dpi. The upper row shows Friesia, and the lower row shows Sebastian Kneipp. Scores were assigned under blue-light illumination in darkness

GFP fluorescence was recorded at 3 and 5 dpi under blue-light illumination (FastGene® Blue/Green LED Flashlight, Nippon Genetics) in a dark room. Expression was scored on a five-class ordinal scale: 0 = no detectable expression, 1 = mild-to-moderate expression, 2 = good expression, 3 = strong expression, and 4 = very strong expression (Fig. 2). The score is interpreted as a semi-quantitative measure of visible transient GFP expression in infiltrated tissue. It does not directly measure bacterial spread, T-DNA copy number or stable transformation efficiency. Three biological replicates per genotype were used for the phenotypic analyses.

### Genotyping, GWAS and candidate-gene annotation

Genotyping data were obtained from the WagRhSNP 68K Axiom SNP array (Koning-Boucoiran et al. 2015). After the marker quality was filtered using a minor allele frequency >= 0.05 and a missing value <= 0.10, 37,161 SNPs were retained for the GWAS. Phenotypic values were Box–Cox transformed before association analysis. GWAS was conducted with GWASpoly, which is designed for autopolyploid species and tests allele-dosage models (Rosyara et al. 2016). Additive and simplex dominance models were evaluated. To control for population structure and relatedness, the model included a kinship matrix using the leave-one-chromosome-out approach and two principal components, following mixed-model GWAS concepts (Yu et al. 2006; Yang et al. 2014). The effective number-of-tests procedure implemented in GWASpoly was used for multiple-testing correction, leading to a genome-wide threshold of –log10(P) = 5.54 (Moskvina and Schmidt 2008). Marker positions were interpreted relative to the *Rosa chinensis* genome assembly (Hibrand Saint-Oyant et al. 2018). SNP contigs within the associated regions were annotated by BLASTx searches against the Genome Database for *Rosa*ceae (GDR) and National Center for Biotechnology (NCBI) protein databases (Jung et al. 2019).

### Statistical analyses

Statistical analyses were performed in R version 4.3.1 (R Core Team 2023). Genotypic differences in GFP expression were tested using the Kruskal–Wallis rank-sum test and analysis of variance. Pairwise comparisons were assessed with Tukey’s post hoc tests where appropriate. Pearson correlation coefficients were calculated between 3- and 5-dpi GFP scores and between GFP expression and previously available fragrance scores from the same association panel. Additional diagnostic and supplementary plots are provided in Online Resource 1.

## Results

### Petals of rose genotypes differ strongly in transient GFP expression

**Fig. 3:**
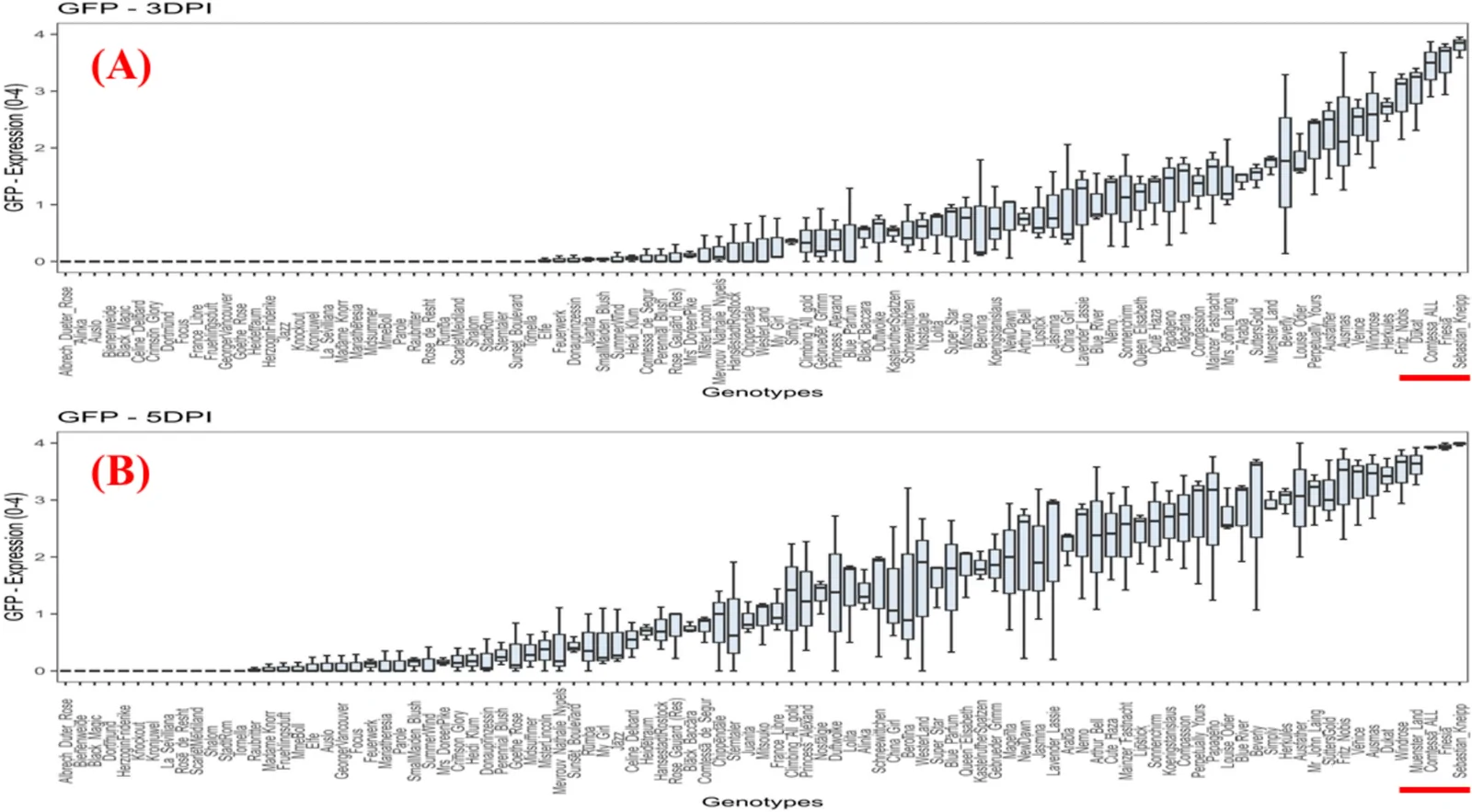
Phenotypic variation for transient GFP expression in 96 rose genotypes. Boxplots show GFP expression scores at 3 dpi (A) and 5 dpi (B), with genotypes arranged by increasing mean score at each time point. Scores range from 0 (no detectable GFP expression) to 4 (very strong GFP expression). Boxes show the first and third quartiles, horizontal lines indicate medians, and whiskers indicate the observed range among the 60 scored petals in the three biological replicates per genotype

Transient GFP expression varied widely among the petals of the 96 rose genotypes. The mean scores ranged from 0 to 3.81 at 3 dpi and from 0 to 3.98 at 5 dpi (Fig. 3). At 3 dpi, the petals of 33 genotypes showed no detectable GFP signal, whereas only 13 genotypes remained at a score of 0 at 5 dpi. The number of genotypes with strong-to-very strong expression increased over time, indicating that 5 dpi provided a more informative endpoint. At 5 dpi, the cultivars Windrose, Münsterland, Comtessa AL, Friesia, and Sebastian Kneipp were among the highest-scoring genotypes, with Sebastian Kneipp reaching a mean score of 3.98. Genotypic effects were highly significant at both time points (3 dpi: Kruskal–Wallis chi-square = 245.94, df = 95, *p* = 2.5e−15; 5 dpi: chi-square = 243.83, df = 95, *p* = 4.814e−15; Online Resource 1, Table S2). Scores at 3 and 5 dpi were strongly correlated (*r* = 0.90, *p* < 0.05; Online Resource 1, Fig. S2), supporting the robustness of the phenotype scoring while justifying the use of the 5-dpi endpoint for GWAS.

### A major association for GFP expression maps to chromosome 2

**Fig. 4:**
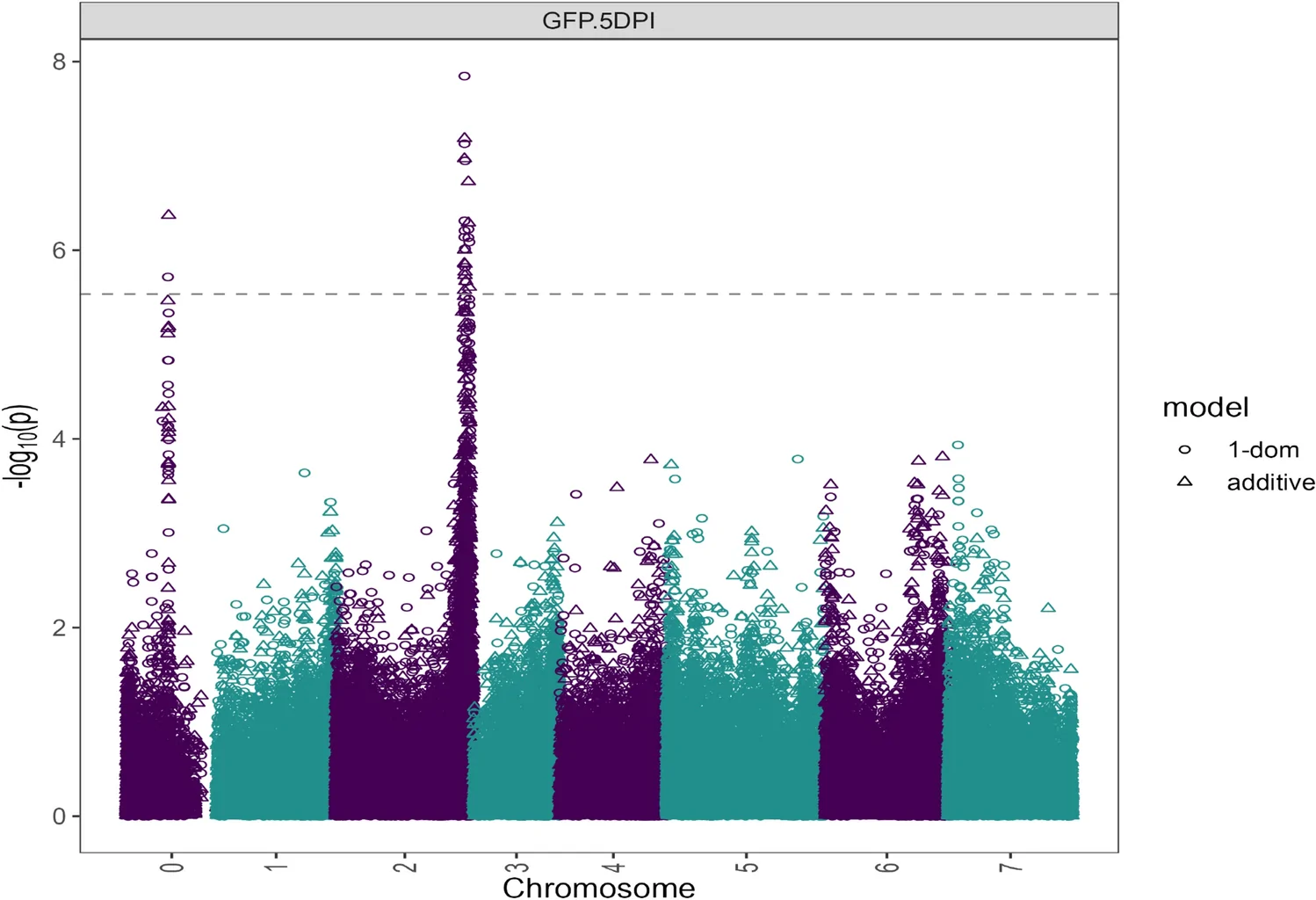
Genome-wide association analysis for GFP expression in petals at 5 dpi. The Manhattan plot shows –log10(P) values for 37,161 SNPs across the *Rosa chinensis* genome, including chromosome 0 contigs not assigned to a defined pseudochromosome. The circles denote the simplex-dominant models, and the triangles denote the additive model. The grey dashed line indicates the M.eff-corrected significance threshold of –log10(P) = 5.54

**Fig. 5:**
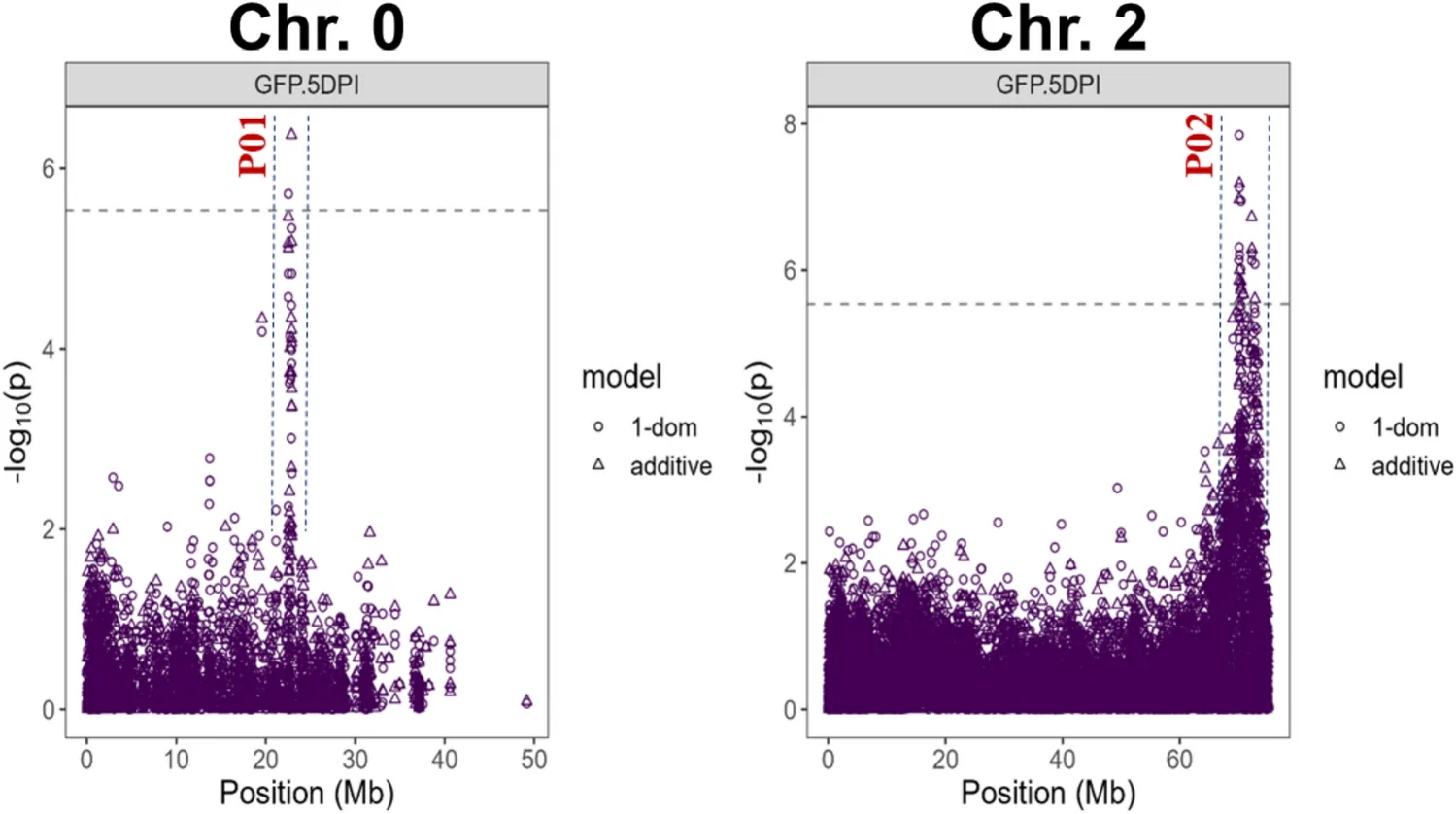
Detailed view of the associated chromosome 0 and chromosome 2 regions. The chromosome 0 peak (P01) is located at approximately 22.5–23.5 Mbp, whereas the chromosome 2 peak (P02) spans approximately 4 Mbp from 69 to 73 Mbp. The grey dashed line indicates the M.eff-corrected significance threshold of –log10(P) = 5.54. The vertical dashed blue lines highlight the significant chromosome 0 and chromosome 2 regions

GWAS of the 5-dpi GFP expression scores revealed a major association on chromosome 2 and a second peak on chromosome 0, which contains contigs not assigned to one of the seven pseudochromosomes (Hibrand Saint-Oyant et al. 2018) (Fig. 4). Q–Q plots indicated acceptable model behaviour, with the strongest deviation from the null expectation corresponding to the associated chromosome 2/0 region (Online Resource 1, Figs. S3, S4). The most significant chromosome 2 marker, RhK5_12481_722P at 70.13 mega base pairs (Mbp), reached –log10(P) = 7.85 under a simplex dominance model. Nineteen significant SNPs were located in the chromosome 2 region, and two significant SNPs were located on chromosome 0. The significant chromosome 2 SNPs covered an approximately 4-Mbp interval from 69 to 73 Mbp (Fig. 5; Table 1). Given the location and co-occurrence of the chromosome 0 peak, these unanchored contigs may represent sequences belonging to the same chromosome 2 region; We therefore blasted the transcript sequences from which 12 of the significant SNPs were developed via NCBI BLAST 2.17.0+ against sequences from Rosa (taxid: 3764) to account for recent updates on genome sequencing in roses (Zhang et al. 2000; Morgulis et al. 2008). When filtered for 100% query coverage and the highest E-values, all 12 sequences matched appropriate positions on chromosome 2 as the strongest hits (data not shown) indicating that this group of SNPs indeed belong to chromosome 2.

**Table 1:** Selected SNP markers associated with transient GFP expression at 5 dpi and candidate-gene annotations

Marker	Peak/Chr	Position (Mbp)	Model	Score	Candidate annotation
RhK5_12481_722P	P02/2	70.13	1-dom-alt	7.85	Unknown protein; thyroid adenoma-associated protein homologue
Rh12GR_23266_2817P	P02/2	70.16	additive	7.19	ABCB19/ABC transporter B family member 19
RhK5_3122_150P	P02/2	70.44	1-dom-ref	6.95	CLP-similar protein 3; CLP1-like pre-mRNA cleavage complex subunit
RhK5_10683_422P	P02/2	72.26	additive	6.73	Unknown protein; proteasome assembly chaperone 3
RhK5_1053_1971P	P02/2	70.13	1-dom-ref	6.31	Cullin 3/cullin-3A
RhK5_18835_67P	P01/00	22.88	additive	6.37	RING/U-box protein with ARM repeat; U-box domain-containing protein 40
RhMCRND_5437_1194P	P02/2	72.29	additive	6.29	Monodehydroascorbate reductase 4, peroxisomal
Rh12GR_92884_1039P	P02/2	70.19	1-dom-alt	6.21	RNA-binding family protein; binding partner of ACD11 1
RhK5_8681_366P	P02/2	70.10	1-dom-ref	6.14	ATP-dependent RNA helicase; DExH-box RNA helicase DExH18
RhMCRND_31882_1184P	P02/2	72.81	1-dom-ref	6.09	Embryo defective 3012; nuclear pore complex protein GP210-like
RhK5_2002_1055P	P02/2	70.40	1-dom-ref	6.02	High-mobility-group protein; FACT complex subunit SSRP1-like
Rh12GR_65803_297P	P02/2	70.42	1-dom-ref	5.82	SPFH/Band 7/PHB domain protein; hypersensitive-induced response protein 1-like
RhK5_14601_1092P	P01/00	22.53	1-dom-ref	5.72	5-prime−3-prime exonuclease family protein; DNA repair protein UVH3-like
Rh12GR_37946_1503P	P02/2	70.85	additive	5.67	No confident annotation
RhK5_13988_1028P	P02/2	70.12	additive	5.57	Unknown protein; thyroid adenoma-associated protein homologue
RhK5_10527_1407P	P02/2	70.47	1-dom-alt	5.51	DUF185 protein; arginine methyltransferase NDUFAF7-like
RhK5_12478_1400P	P02/2	72.72	1-dom-alt	5.48	ARM repeat superfamily protein; RST1-like protein
RhMCRND_1413_121P	P02/2	69.77	1-dom-alt	5.43	Granule-bound starch synthase 2-like
RhK5_2606_2290P	P02/2	72.81	1-dom-ref	5.42	Embryo defective 3012; nuclear pore complex protein GP210-like
Rh12GR_23266_912P	P02/2	70.16	1-dom-ref	5.35	ABCB19/ABC transporter B family member 19
Rh12GR_849_1041P	P02/2	70.10	1-dom-alt	5.34	ATP-dependent RNA helicase; DExH-box RNA helicase DExH18

Scores are –log10(P). The significance threshold was –log10(P) = 5.54. Candidate annotations were inferred from BLASTx searches against the GDR and NCBI protein databases and should be verified experimentally

### Candidate genes in the associated region

The 21 selected associated SNPs from the chromosome 0 and 2 regions were located within or near genes with diverse predicted functions (Table 1). Several annotations are plausible for host control of transient expression and are potential candidate genes. The chromosome 2 peak included SNPs in or near an ABC transporter B family member 19 homologue, a Cullin 3 homologue, DExH-box RNA helicase candidates, an RNA-binding protein, a hypersensitive-induced response protein 1-like membrane-associated protein, monodehydroascorbate reductase 4, and nuclear pore complex protein GP210-like sequences and uncharacterised proteins. Because the local linkage disequilibrium (LD) in the panel decayed over megabase-scale distances (Online Resource 1, Fig. S5), multiple linked genes may contribute to the signal, and the associated interval should be treated as a candidate region rather than a single-gene result.

### Allele dosage at representative SNPs separates high and low GFP expression classes

**Fig. 6:**
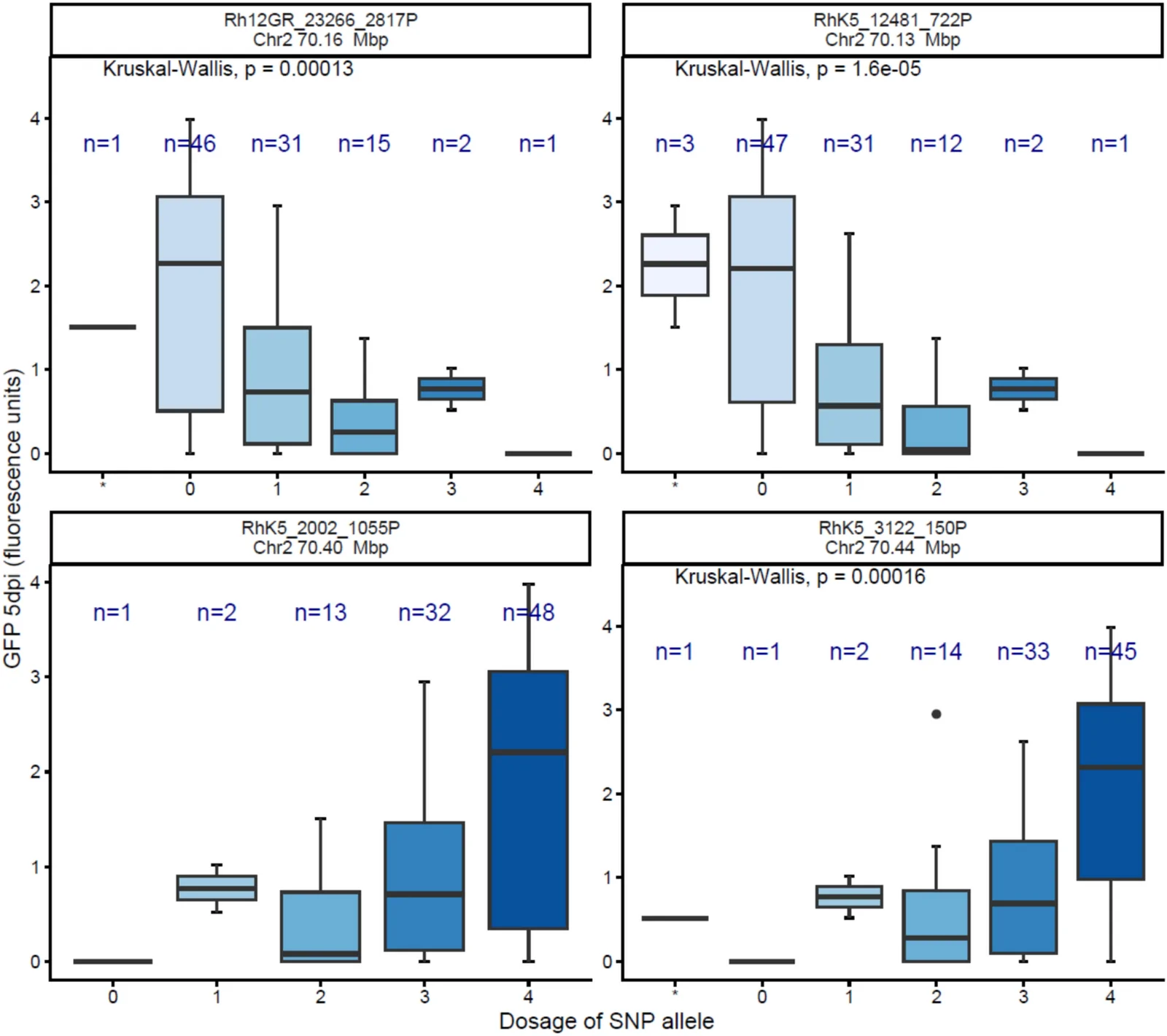
Allele-dosage effects of four representative SNPs in the chromosome 2 peak. Boxplots showing GFP expression at 5 dpi for the Rh12GR_23266_2817P, RhK5_12481_722P, RhK5_2002_1055P, and RhK5_3122_150P alleles. The numbers above the boxes indicate the number of genotypes per class. Asterisks indicate genotypes for which the dosage classes could not be determined with the respective SNP. Kruskal–Wallis *p* values are shown within the panels. Comparison with fragrance phenotypes suggests linked but distinct traits

Allele-dosage plots for representative markers revealed strong quantitative marker effects within the chromosome 2 region (Fig. 6). For Rh12GR_23266_2817P and RhK5_12481_722P near 70.13–70.16 Mbp, genotypes carrying a low allele dosage had substantially higher median GFP scores than the other dosage classes did. Conversely, for RhK5_3122_150P and RhK5_2002_1055P, the opposite homozygous or quadruplex dose class presented the highest GFP expression (see Online Resource 1 for details, Fig. S6A, B, C). The allele-class distribution was uneven for some markers, as expected in a heterogeneous tetraploid panel. For two of these SNPs (Rh12GR_23266_2817P and RhK5_2002_1055P), 25 of the 46 cultivars in the high-expression dosage classes reached GFP scores >= 2, corresponding to 54.3% of the cultivars in those classes (Online Resource 1, Fig. S7).

The chromosome 2 region overlaps with marker clusters previously observed for fragrance-related variation in the same rose association panel (Online Resource 1, Fig. S8). A direct comparison of GFP expression and fragrance, however, revealed only a weak positive correlation (*r* = 0.27, *p* < 0.05) (Online Resource 1, Fig. S9), explaining less than 8% of the phenotypic variance. The distributions of both traits also differed substantially (Online Resource 1, Figs. S10 and S11). These results may indicate regulation by different linked loci in the same broad genomic region rather than a single gene controlling both fragrance and transient GFP expression (Online Resource 1, Fig. S12).

## Discussion

### Genotype-dependent transient expression provides a practical rose screening trait

This study demonstrated strong genotype-dependent variation in transient GFP expression after *Agrobacterium* infiltration of rose petals. The highest-scoring genotypes, especially the Sebastian Kneipp, Friesia, and Comtessa AL cultivars, are immediate candidates for functional assays in petals. Because stable transformation of rose is difficult, time-consuming and genotype-dependent, identifying permissive genotypes for transient assays can accelerate early stage gene function tests before stable transformation or genome editing is attempted. The high correlation between the 3- and 5-dpi scores indicates that the assay is robust, whereas the broader dynamic range at 5 dpi makes this endpoint preferable for genetic mapping and genotype selection.

The chromosome 2 region is a major locus for transient GFP expression The GWAS detected a major region on chromosome 2 that explained a substantial part of the genotype-dependent response. The presence of a related peak on chromosome 0 suggests that unanchored contigs may belong to the same physical region, although this should be verified with improved genome anchoring. Our association panel with only 96 plants very much limits the resolution of the GWAS study. Therefore, the broad interval from 69 to 73 Mbp is compatible with the LD pattern observed in the panel and with the limited mapping resolution expected from only 96 genotypes. Therefore, the results should not be interpreted as evidence for a single causal gene at this time. Instead, the region represents a high-priority interval containing a large number of candidates whose functions could influence T-DNA delivery, transient expression, tissue competence, or defence responses. Future studies might use a larger panel to narrow this interval and reduce the number of candidate genes, as functional studies, including stable transformation in roses, are laborious, as mentioned before. In addition, GWAS in larger panels combined with sequencing the alleles of candidate genes may allow the identification of particular genetic variants correlated with the permissiveness of transient transformation with sufficient statistical power.

Candidate genes point to host processes relevant to *Agrobacterium-*mediated expression. Several candidate annotations in the associated region are biologically plausible. Cullin-related proteins and the ubiquitin–proteasome system have been implicated in *Agrobacterium*–host interactions, including the removal of T-complex-associated proteins and the modulation of host responses (Lacroix et al. 2006; Hwang et al. 2017). RNA helicases, RNA-binding proteins and CLP1-like proteins can influence the expression or processing of delivered transcripts. Nuclear pore complex GP210-like annotations are also noteworthy, because nuclear import is an essential step between T-DNA delivery and reporter expression. The ABCB19 candidate is involved in auxin transport in *Arabidopsis* and functions in postembryonic organ separation (Zhao et al. 2013), which may be relevant to petal tissue physiology, wound responses, or cell competence. Defence-related annotations, including hypersensitivity-induced response protein-like genes, are also plausible, because plant immune signalling can restrict *Agrobacterium*-mediated transformation; for example, an MKK4/5-MPK3/6 cascade modulates transformation by regulating immunity in *Arabidopsis* (Liu et al. 2021). These candidates require validation by expression analysis, sequencing of contrasting genotypes, and functional assays.

### Assay interpretation and limitations

The phenotype measured here is visible transient GFP expression, not bacterial migration or stable transformation. Low GFP expression could result from reduced bacterial entry, impaired T-DNA transfer, weak transient expression, rapid silencing, tissue necrosis, promoter differences, cell wall or cuticle properties, or defence activation. Conversely, high expression does not guarantee efficient stable transformation or regeneration. The ordinal scoring system was sufficiently robust to reveal a major quantitative trait locus (QTL), but future work should include quantitative image analysis of fluorescence area and intensity to improve the resolution of phenotyping, histological assessments of petal tissue structure, and direct measurement of bacterial load or T-DNA abundance. Increasing the panel size and using updated rose genome assemblies would improve mapping resolution. The strongest candidate genes should be tested by sequencing and expression analysis in contrasting genotypes, followed by transient overexpression, silencing, or second-transformation assays in petals or rose hairy roots.

## Conclusion

A diverse rose panel revealed extensive genetic variation for transient GFP expression in petals after *Agrobacterium* infiltration. GWAS revealed a major chromosome 2 region, accompanied by a chromosome 0 peak likely related to the same genomic interval. The associated markers and high-expression genotypes provide immediate resources for rose functional genomics, whereas candidate genes in the interval offer testable hypotheses about host control of transient *Agrobacterium*-mediated expression.

## Supplementary Information

Below is the link to the electronic supplementary material.

Supplementary file1 (DOCX 2303 kb)

## Data Availability

Original data will be made available after publication of the manuscript under the following web address: https://doi.org/10.25835/g1xnmiik.
