# Supplementary material for: A chromosome 2 locus controls transient Agrobacterium-mediated GFP expression in rose petals
Source: Plant Cell Rep. 2026 Sep 4;45(9):278. doi: 10.1007/s00299-026-03961-z (PMC13545033; doi:10.1007/s00299-026-03961-z)
Supplement: Supplementary file 1 [file 299_2026_3961_MOESM1_ESM.docx]

**Electronic supplementary material**

Online Resource 1

Journal: Plant Cell Reports

Article title: A chromosome 2 locus controls transient *Agrobacterium*-mediated GFP expression in rose petals

Authors: Ninh Hai Ho, Marcus Linde, Thomas Debener

Corresponding author: [Thomas Debener, debener@genetik.uni-hannover.de, ORCID: 0000-0002-2012-2246] [Name, e-mail, ORCID]

# Supplementary figures

Figure S1. Binary vector C757pGFPU10-35s-ocs-LH (DNA Cloning Service, Hamburg, Germany) used for Agrobacterium-mediated transient expression assays. The T-DNA carries an intron-containing GFP reporter driven by the Arabidopsis ubiquitin 10 promoter. The map was created using the software SnapGene Viewer.

Figure S2. Pearson correlation between GFP expression score at 3 dpi and 5 dpi across rose genotypes. The strongly positive association (r = 0.90, p < 0.05) supports linked the ability of agrobacteria to infiltrate the rose petals at both time points the significant differences

Figure S3. Q-Q plots for GWAS models of transient GFP expression at 3 dpi. Additive, 1-dom-ref and 1-dom-alt models were evaluated by chromosome. The strongest deviations from the null expectation correspond to the chromosome 2/chromosome 00 association region.

Figure S4. Q-Q plots for GWAS models of transient GFP expression at 5 dpi. Additive, 1-dom-ref and 1-dom-alt models were evaluated by chromosome. The strongest deviations from the null expectation correspond to the chromosome 2/chromosome 00 association region.

Figure S5. Genome-wide linkage disequilibrium decay in the 96-genotype rose association panel. Pairwise LD (r2) was plotted against physical distance for filtered SNPs. Average LD decreased below approximately r^2^ = 0.04 within about 15 Mbp and below r^2^ = 0.02 within about 46 Mbp.

Figure S6A. Allele-dosage boxplots for 21 SNP markers associated with transient GFP expression at 5 dpi.

Figure S6B. Allele-dosage boxplots for 21 SNP markers associated with transient GFP expression at 5 dpi.

Figure S6C. Allele-dosage boxplots for 21 SNP markers associated with transient GFP expression at 5 dpi.

Figure S7. Percentage of cultivars with GFP expression score >= 2 in allele dosage classes for representative chromosome 2 SNPs. Values above bars indicate the percentage of high-expression cultivars in each class.

Figure S8. Comparison of association profiles for fragrance-related variation (Schulz et al. 2021) and transient GFP expression in the rose association panel. The overlapping chromosome 2 region suggests physical proximity of trait-associated loci, but direct phenotypic correlation is weak.

Figure S9. Pearson correlation between fragrance score and GFP expression at 5 dpi across rose genotypes. The weak positive association (r = 0.27, p < 0.05) supports linked but largely distinct genetic regulation.

Figure S10. Allele-dosage effects of representative chromosome 2 markers on fragrance and transient GFP expression. The figure illustrates that some marker classes show similar trends for both traits, but this does not establish a shared causal gene.

Figure S11. Phenotypic distributions of GFP expression at 5 dpi and fragrance score in the association panel. Both traits are scored from 0 to 4, but their distributions differ.

Figure S12. Detailed GLM-based view of SNP associations in the chromosome 2 interval. Seven local peaks are visible between approximately 70 and 73 Mbp, suggesting that several linked genes may contribute to the broad association region.

# Supplementary tables

Table S1. Composition of the culture media and antibiotics used for culturing *A. tumefaciens*.

| **Medium** | **Composition** | **Weight/L (g)** |
| --- | --- | --- |
| **YEP liquid** | Peptone/Tryptone | 10 |
|  | Yeast extract | 10 |
|  | Sodium chloride (NaCl) | 5 |
|  | Adjust pH to 7.5 |  |
|  | *Rifampicin (50μg/mL) | 1000 μl/L |
|  | *Streptomycin (50μg/mL) | 1000 μl/L |
|  | *Spectinomycin (50μg/mL) | 1000 μl/L |
| Note: * Selective antibiotic is added as recommended. YEP medium is subject to sterilization by autoclaving. | | |

Table S2. Statistical summary of genotype effects for transient GFP expression.

| **Trait/time** | **N genotypes** | **Kruskal-Wallis chi-square** | **df** | **p value** | **ANOVA F** | **ANOVA df** | **Partial eta squared** | **Model R2** |
| --- | --- | --- | --- | --- | --- | --- | --- | --- |
| GFP, 3 dpi | 96 | 245.94 | 95 | 2.5e-15 | 13.481 | 95,192 | 0.870 | 0.870 |
| GFP, 5 dpi | 96 | 243.83 | 95 | 4.814e-15 | 11.007 | 95,192 | 0.845 | 0.845 |

**Note.** Values are derived from the chapter statistical output. The ANOVA statistics are presented as descriptive support for genotype effects; because the GFP score is ordinal and non-normally distributed, the Kruskal-Wallis test is the primary inferential test.
